# Initial commented checklist of Iranian mayflies, with new area records and description of *Procloeon caspicum* sp. n. (Insecta, Ephemeroptera, Baetidae)

**DOI:** 10.3897/zookeys.749.24104

**Published:** 2018-04-10

**Authors:** Jindřiška Bojková, Pavel Sroka, Tomáš Soldán, Javid Imanpour Namin, Arnold H. Staniczek, Marek Polášek, Ľuboš Hrivniak, Ashgar Abdoli, Roman J. Godunko

**Affiliations:** 1 Department of Botany and Zoology, Masaryk University, Kotlářská 2, CZ-61137 Brno, Czech Republic; 2 Biology Centre, Czech Academy of Sciences, Institute of Entomology, Branišovská 31, CZ-37005 České Budějovice, Czech Republic; 3 Department of Fishery, Faculty of Natural Resources, University of Gilan, POB 1144, Sowmehsara-Rasht, Iran; 4 Department of Entomology, State Museum of Natural History Stuttgart, Rosenstein 1, 70191 Stuttgart, Germany; 5 Department of Biodiversity and Ecosystem Management, Environmental Sciences Research Institute, Shahid Beheshti University, Daneshjou Boulevard,1983969411 Tehran, Iran; 6 Faculty of Sciences, University of South Bohemia, Branišovská 31, CZ-370 05 České Budějovice, Czech Republic; 7 State Museum of Natural History, National Academy of Sciences of Ukraine, Teatralna 18, UA-79008, Lviv, Ukraine

**Keywords:** aquatic biodiversity, biogeography, faunistic research, Middle East, taxonomy

## Abstract

An initial checklist of mayflies (Ephemeroptera) of Iran is compiled based on critical review of available literature data, complemented with new data from 38 localities of Gilan and Ardabil provinces. At present, altogether only 46 species and 25 genera are known from Iran, 18 species are reported as new to Iran in this study. Some previously published data are critically evaluated and doubtful taxa are excluded from the list. Basic analysis of the distribution and biogeography of recorded species is given. Procloeon (Pseudocentroptilum) caspicum Sroka, **sp. n.** is described based on mature larva and egg. Critical differential diagnostic characters distinguishing the species from related taxa are discussed in detail.

## Introduction

In comparison to Europe, the mayfly fauna of the Middle East is less known and data from some regions are still fragmentary. Extensive research on mayflies has been mainly focused on the Arabian Peninsula (Thomas and Sartori 1989, Sartori and Gillies 1990, Sartori 1991, Gattolliat and Sartori 2008) and neighbouring countries, namely Syria and Lebanon (Koch 1980, 1981, 1988, Thomas and Dia 1982, 1983, 1984, 1985, 1999, 2007, Thomas et al. 1988, 2007), Jordan (Gattolliat et al. 2012), and Israel (Demoulin 1973, Malzacher 1992, Sartori 1992, Yanai et al. 2017). Extensive literature is available from Turkey (for a review see Kazancı and Türkmen 2012 and Salur et al. 2016). In contrast, Iran, Iraq, Afghanistan, and Pakistan have been poorly investigated and only random findings of mayflies have been published to date (e.g., Kimmins 1950, Demoulin 1964, Allen 1973, Al-Zubaidi et al. 1987, Bojková and Soldán 2015).

Iran, the second largest (more than 1.6 million km²) country of the region after Saudi Arabia, has been studied only occasionally so far. Only 19 species of mayflies have been reported in 16 short taxonomic contributions published in international entomological journals. They include mostly simple faunistic records of species already known from neighbouring countries (Tshernova 1949, Soldán and Landa 1977, Braasch 1981, Kluge 1987, Jacobus 2009, Soldán and Godunko 2013, Godunko et al. 2017). Descriptions of new species were often based on few specimens, often of a single developmental stage (Soldán 1978a, Braasch and Soldán 1979, Braasch 1981, 1983a,b, Sartori and Sowa 1992, Jacobus et al. 2009). The vast majority of records are limited to the northern part of Iran (mostly Alborz Mts. and its surroundings). The only comprehensive study of Iranian mayflies is a monography by Mohammadian (2005). It is written in Persian, thus inaccessible for a wider scientific audience. Moreover, it does not include new records, but is a mere compilation of literature without any own data contributed by the author. It enumerates 55 mayfly species presumably occurring in Iran. However, the list includes species reported from the Iranian Plateau, an area roughly extending from Tigris River to Indus River, which not only comprises Iran, but also some parts of neighbouring countries, Iraq, Azerbaijan, Turkmenistan, Afghanistan and Pakistan. Consequently, a significant part of the species listed should not be regarded as valid records actually documenting the occurrence of species in Iran unless being further corroborated.

Other sources of information on the mayfly diversity in Iran are some ecological studies on benthic macroinvertebrates over the last decades in order to assess water quality. Sharifinia (2015) reviewed 57 references (37 of them written in Persian) published after 2000 and compiled a list of 37 mayfly taxa (identified to species or genus level) known from Iranian rivers. However, this list is partially based on studies presenting species/genera which identity should be regarded as highly unlikely (Ahmadi et al. 2011, 2012, Mahboobi Soofiani et al. 2012, Amri et al. 2014, Farasat and Sharifi 2014, Golchin Manshadi et al. 2015). They list as many as 27 taxa (species or genera) known exclusively from the Nearctic and Neotropic Regions, the occurrence of which can be definitively excluded in the Middle East. Therefore, Sharifinia’s as well as Mohammadian’s lists of mayfly species should not be regarded as reliable, thus maintaining a significant gap in our knowledge. The area of Iran should be viewed a certain transitory zone hosting West Palaearctic (European) and Caucasian elements of fauna on one hand and Central Asian or even Oriental faunal elements on the other being certainly worth of the detailed and extensive study. Controversial or irrelevant distributional data should be deleted from faunistic lists, and existing data should be critically evaluated based on extensive, large-scale collecting of new material at localities covering the entire territory of Iran. The *sine qua non* condition is proper species identification even if requiring taxonomic revision of some taxa.

As a first step to achieve this ambitious goal, in May 2016 we collected mayflies at 38 localities in the Gilan and Ardabil Provinces in the north western Iran, the areas of presumably high Ephemeroptera diversity. This study represents the first part in a series of contributions aiming to provide a realistic and more complex picture of the Iranian mayfly fauna for future reference. Hence, the objective of the present study is to (i) critically review all mayfly records so far published from Iran with an emphasis on the validity of species occurrence in Iran and status of species from the taxonomic point of view, (ii) provide new data on the occurrence of species in the western part of the Caspian Sea region, and (iii) describe a new species of the genus *Procloeon* recently found in the studied area.

## Study area

**Figure 1.**
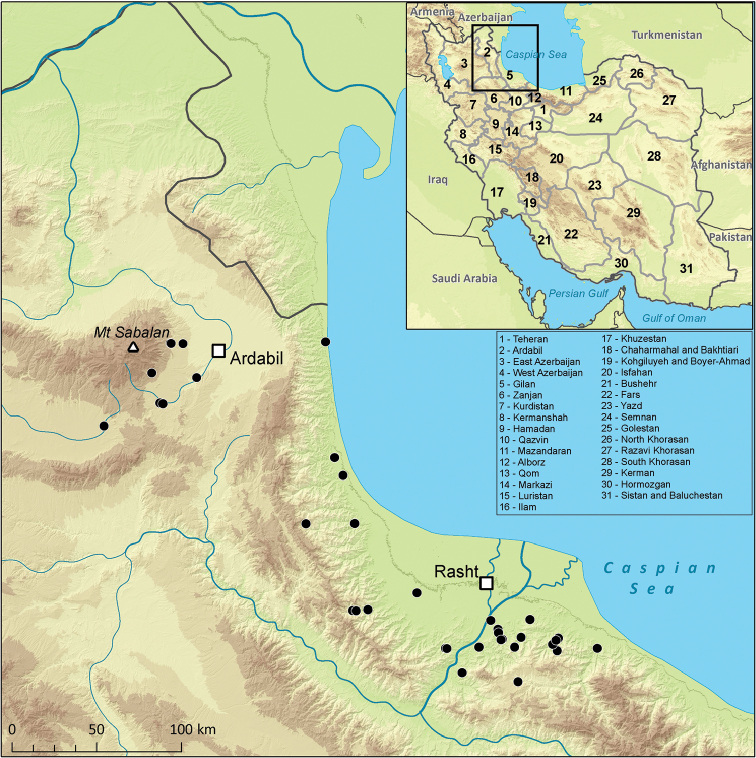
Map of the localities sampled in May 2016 with a list of provinces of Iran.

Mayflies were collected at 38 localities in the Gilan and Ardabil Provinces in 2016 (Fig. 1). Studied localities included four types of landscape common at the region. (i) Five streams in the Caspian Sea coastal plains in the Gilan Province at the altitude from -6 to 40 m a.s.l. (localities No. 5, 13, 27, 36, 37 in Table 1). The Caspian Sea coastal plains are almost entirely covered by agricultural and urban land. Studied streams drained substantial area of rice fields and a mosaic of various croplands. Stream network of the Caspian Sea coastal plains was modified by numerous channels irrigating fields and interconnections of streams enabling needed distribution of water. Streams are often impacted by pollution from intensive agriculture and urban sewage. (ii) In total, 22 streams flow in the humid deciduous broadleaved forest at the northern slopes of the western part of the Alborz Mts. The altitude of the localities ranged from 80 to 820 m a. s. l. and they include relatively untouched, rapid, turbulent mountain streams (localities No. 6, 11, 15, 17, 32 in Table 1), shaded small, clear brooks (localities No. 2, 7, 8, 10, 16, 28, 30, 31, 33, 34), and eutrophic streams influenced by agriculture and settlements in the river valleys (localities No. 1, 3, 4, 9, 18, 35). (iii) Three streams (localities No. 12, 14, 29) were sampled above 1000 m a.s.l. in the Gilan Province and (iv) eight streams and one pond in the Ardabil Province (altitude 1430–2240 m a.s.l., localities No. 19–26, 38). Localities in the Ardabil Province included only streams in the Sabalan Mt. (4811 m a.s.l.) environ. This region is prone to very extensive agricultural exploitation.

**Table 1.** List of localities studied in the Gilan and Ardabil Provinces in May 2016 (RT – right tributary, LT – left tributary).

Stream types	Site no.	Stream name	Stream	Location	Nearest town	Latitude	Longitude	Altitude	Sampling date
rivers in the coastal plains	5	Sefid Rud	about 55 km from its mouth	SE of Sangar	Sangar	37°07'16"N, 49°44'06"E		39	12/05/2016
rivers in the coastal plains	13	Shakhzar	about 32 km from its mouth	NE of Fuman	Fuman	37°14'13"N, 49°20'43"E		5	15/05/2016
rivers in the coastal plains	27	Chelvand	about 2.5 km from its mouth	W Chelvand (S of Lavandvil)	Lavandvil	38°17'20"N, 48°51'35"E		-6	19/05/2016
rivers in the coastal plains	36	Karganrud	about 7 km from its mouth	in Talesh	Talesh	37°48'22"N, 48°54'27"E		36	22/05/2016
rivers in the coastal plains	37	Navrud	about 5 km from its mouth	in Asalem	Asalem	37°43'56"N, 48°57'13"E		34	22/05/2016
clear forest rivers	6	Zilaki River	RT of Sefid Rud	in Mush Bijar (E of Shahr-e Bijar)	Shahr-e Bijar	37°00'28"N, 49°40'24"E		125	13/05/2016
clear forest rivers	11	Shafa Rud	about 20 km from its mouth	W of Punel	Punel	37°31'47"N, 49°00'52"E		218	15/05/2016
clear forest rivers	15	Machian	LT of Bala Rud	Lunak waterfalls (S of Siahkal)	Siahkal	37°00'31"N, 49°51'51"E		484	16/05/2016
clear forest rivers	17	Shamrud	RT of Sefid Rud	south of Tushi (S of Siahkal)	Siahkal	37°03'00"N, 49°53'54"E		252	16/05/2016
clear forest rivers	32	Semoosh	RT of Polrud	SW of Rahimabad	Rahimabad	37°00'11"N, 50°18'06"E		88	21/05/2016
clear forest brooks	2	Eshkaraab	RT of Khara Rud	S of Paein Khara Rud (S of Pashaki)	Sangar	37°02'29"N, 49°47'52"E		198	12/05/2016
clear forest brooks	7	Sefidab	RT of Siah Rud	in Divarsh (NE of Shirkuh)	Tutkabon	36°53'59"N, 49°35'06"E		273	13/05/2016
clear forest brooks	8	Chulak waterfall	LT of Reshte Rud	NE of Khulak (W of Oskolak)	Oskolak	37°00'11"N, 49°29'49"E		201	13/05/2016
clear forest brooks	10	Sangdeh	LT of Shafa Rud	W of Punel	Punel	37°31'47"N, 49°00'52"E		218	15/05/2016
clear forest brooks	16	unnamed brook	Lunak waterfalls	Lunak waterfalls (S of Siahkal)	Siahkal	37°00'31"N, 49°51'49"E		495	16/05/2016
clear forest brooks	28	unnamed brook	LT of Shalman Rud1	SW of Amlash	Amlash	37°02'46"N, 50°05'42"E		184	21/05/2016
clear forest brooks	30	unnamed brook	RT of Shalman Rud	in Bolurdekan	Amlash	37°01'55"N, 50°04'39"E		282	21/05/2016
clear forest brooks	31	unnamed brook	LT of Shalman Rud2	SW of Amlash	Amlash	37°02'13"N, 50°04'57"E		287	21/05/2016
clear forest brooks	33	unnamed brook	LT of Rudkhan	NE of Masuleh	Masuleh	37°09'47"N, 49°00'17"E		820	22/05/2016
clear forest brooks	34	unnamed brook	RT of Rudkhan	NE of Masuleh	Masuleh	37°09'42"N, 49°01'17"E		697	22/05/2016
polluted forest rivers	1	Khara Rud	RT of Sefid Rud	S of Paein Khara Rud (S of Pashaki)	Sangar	37°05'01"N, 49°46'25"E		81	12/05/2016
polluted forest rivers	3	Kalardeh Rukhan	left fork of Khara Rud	in Madarsara (S of Pashaki)	Sangar	37°04'12"N, 49°46'36"E		103	12/05/2016
polluted forest rivers	4	unnamed brook	right fork of Khara Rud	in Golestansara (S of Pashaki)	Sangar	37°02'20"N, 49°47'27"E		186	12/05/2016
polluted forest rivers	9	Reshteh Rud	LT of Sefid Rud	NE of Khulak (W of Oskolak)	Oskolak	37°00'07"N, 49°30'13"E		185	13/05/2016
polluted forest rivers	18	Choshal		E of Ezbaram (S of Lahijan)	Lahijan	37°07'33"N, 49°56'39"E		146	16/05/2016
polluted forest rivers	35	Masuleh Rudkhan	about 50 km from its mouth	E of Masuleh	Masuleh	37°10'02"N, 49°05'03"E		369	22/05/2016
streams above 1000 m in Gilan Prov.	12	unnamed brook	LT of Shafa Rud	NW of Sangdeh	Sangdeh	37°31'46"N, 48°45'19"E		1337	15/05/2016
streams above 1000 m in Gilan Prov.	14	Kakrud	LT of Polrud	in Ishku-ye Bala (SW of Deylaman)	Deylaman	36°51'44"N, 49°52'52"E		1356	16/05/2016
streams above 1000 m in Gilan Prov.	29	unnamed brook		N of Chaldasht	Amlash	36°59'86"N, 50°05'73"E		1250	21/05/2016
streams above 1000 m in Ardabil Prov.	19	unnamed brook	small brook below Alvares ski areal	in Alvaresi (W of Sarein)	Sarein	38°09'38"N, 47°56'21"E		2237	17/05/2016
streams above 1000 m in Ardabil Prov.	20	Bulakhlar chayi	left fork of the river	NW of Nir	Nir	38°02'09"N, 47°58'55"E		1622	17/05/2016
streams above 1000 m in Ardabil Prov.	21	Bulakhlar chayi	LT of Hakim Geshlaghi chayi	NW of Nir	Nir	38°02'09"N, 47°58'55"E		1622	17/05/2016
streams above 1000 m in Ardabil Prov.	22	unnamed brook		in Sardabe (W of Vakilabad)	Vakilabad	38°17'03"N, 48°02'10"E		1927	18/05/2016
streams above 1000 m in Ardabil Prov.	23	unnamed brook		below Sardabe (W of Vakilabad)	Vakilabad	38°16'58"N, 48°02'28"E		1901	18/05/2016
streams above 1000 m in Ardabil Prov.	24	Hakim Geshlaghi chayi	RT of Qareh-Su	SW of Almas (SW of Ardabil)	Ardabil	38°08'27"N, 48°10'36"E		1433	18/05/2016
streams above 1000 m in Ardabil Prov.	25	Bulakhlar chayi	LT of Hakim Geshlaghi chayi	in Nir	Nir	38°01'56"N, 47°59'48"E		1602	18/05/2016
streams above 1000 m in Ardabil Prov.	26	unnamed brook		E of Kadijan (E of Sarab)	Sarab	37°56'25"N, 47°41'03"E		1717	18/05/2016
streams above 1000 m in Ardabil Prov.	38	pond	pond on brook 22 & 23	SE of Jomadi (E of Vakilabad)	Vakilabad	38°16'55"N, 48°06'14"E		1589	18/05/2016

Studied localities belong to the Euxino-Hyrcanian Province of the Euro-Siberian subregion of the Palaearctic Region (Sagheb Talebi et al. 2014). The climate is very humid, with cold winters, without dry period (annual precipitations 2000 mm, annual mean temperature 15 °C) in the western part of the Province and humid with mild winters and short dry period (annual precipitations 600 mm, annual mean temperature 18 °C) in its eastern part. The growing season lasts 7–9 months (Sagheb Talebi 2005, Sagheb Talebi et al. 2014). The Euxino-Hyrcanian Province is famous for its Hyrcanian and Arasbaran forest zones (Sagheb Talebi et al. 2014). The Hyrcanian Forest contains remnants of the broad leaf forests that covered most of the North Temperate Zone in the early Cenozoic (25–50 million years ago), as it was little impacted by Pleistocene climatic changes. Among 65 tree species known from the Hyrcanian Forest, there are several Tertiary relict species such as Caucasian zelkova *Zelkova carpinifolia*, Persian ironwood *Parrotia persica*, and Caucasian walnut *Pterocarya fraxinifolia*. Due to high humidity, the Hyrcanian Forest hosts many epiphytes, mosses, ferns, lichens, mistletoes, and flowering plants (greenbriar *Smilax excelsa* and ivy *Hedera pastuchovii*). It is also characterized by the lack of conifers (except for, e.g., European yew *Taxus baccata*, Junipers, and Mediterranean Cypress Cupressus sempervirens var. horzontalis) (Sagheb Talebi 2005, Sagheb Talebi et al. 2014). The *Querco*-*Buxetum* forests of the Caspian coastal plains have been almost completely converted to agricultural land. On the relatively less humid lower slopes of the mountains (below 700 m a.s.l.) in Gilan and Mazandaran provinces, chestnut-leaved oak (*Quercus castaneifolia*) and European hornbeam (*Carpinus betulus*) are mixed with Persian ironwood forming diverse *Querco*-*Carpinetum* and *Parrotio*-*Carpinetum* forests. These forests have been extensively exploited. Between 700–1500 m a.s.l., oriental beech (*Fagus orientalis*) is the dominant tree forming the *Fagetum hyrcanum* community, the most diverse and productive forest in the region, which is linked with European beech forests (Sagheb Talebi et al. 2014). Above the beech belt, Caucasian oak and Oriental hornbeam occur up to the timberline at approx. 2700 m a.s.l., forming the *Querco macranthero*-*Carpinetum orientalis* community (Sagheb Talebi 2005, Sagheb Talebi et al. 2014).

## Materials and methods

Published records of mayfly species/genera in Iran were excerpted from available literature and summarised in Table 2. System and nomenclature of Palaearctic species included in Table 2 mostly follow Bauernfeind and Soldán (2012), with some exceptions (classification of Kluge and Novikova (2014) is used for the genus *Nigrobaetis*, Jacobus and McCafferty (2008) for the family Ephemerellidae). Generic and species names of Nearctic/Neotropic species mentioned in ecological studies of Iranian freshwaters were presented in the original form, later taxonomic or nomenclatoric changes were not taken into account.

**Table 2.** Commented list of Ephemeroptera of Iran with notes to their distribution. Species representing new area records to Iranian mayfly fauna are **in bold**, data on taxa with unlikely occurrence are marked with an asterisk (*). See Table 1 for numbers and for precise location of localities in the Gilan and Ardabil Provinces studied recently. Basic information on the area of species is based on Bauernfeind and Soldán (2012), detailed information concerning the Middle East and Central Asia is provided by references. Data on the occurrence of solely Nearctic/Neotropic species and genera are mentioned below the table (^#^).

Species/genus	Records from Iran	Notes to the global area and distribution	Remarks to records in Iran
Ameletidae			
*Ameletus sp.	Qazvin Prov.: Shahrud	Holarctic genus, with the area extension to Central America and Oriental Region. The only Euro-Siberian species is *A. inopinatus* Eaton, 1887, in Central Asia *A. alexandrae* Brodsky, 1930.	Unidentified species reported by Sharifinia et al. (2016a, b). The nearest record of the genus (*Ameletus inopinatus*) was published from Turkey.
Siphlonuridae			
*Siphlonurus sp.	Isfahan Prov.: Zayanderud,	Holarctic genus, including subarctic areas. Twelve species known from the West Palearctic Region.	Unidentified species reported by Mahboobi Soofiani et al. (2012).
Ametropodidae			
*Ametropus* sp.	„117 km south of Rasht“	Holarctic genus. In the West Palearctic Region, the only species *Ametropus fragilis* Albarda, 1878. *Ametropus eatoni* Brodsky, 1930 described from Siberia, Ural requires re-evaluation.	Unidentified species reported from unclear locality 117 km S of Rasht by Braasch (1981). Family Ametropodidae reported from the unnamed stream NW of Shiraz by Bashti and Ostovan (2014). The nearest record of the genus (*Ametropus fragilis*) was published from the Caucasus (Eaton 1883–1888, Sadovsky 1940).
Baetidae			
Baetis (Acentrella) sp.	Isfahan Prov.: Zayanderud	Holarctic and Oriental genus. Five species known from the West Palearctic Region, additional species known from Central Asia.	Unidentified species reported by Mahboobi Soofiani et al. (2012).
*Baetis* sp.	Qazvin Prov.: Shahrud; Alborz Prov.: Kordan riv., Haraz riv., Tehran Prov.: Jajrud, Bareghan riv.; Mazandaran Prov.: Tajan riv., Dalir riv., Chatan riv., Firuz Abad riv.; Ardabil Prov.: Gharasou riv.; Kermanshah Prov.: Kavat riv.; Isfahan Prov.: Zayanderud; and 50 km SE of Khorramabad, 1500 m a.s.l.	Cosmopolitan genus except for South America. Very diverse in the West Palaearctic Region, at least 64 species known from Europe.	Unidentified species reported by Sharifinia et al. (2016a,b; Shahrud), Mousavi Nadushan and Ramezani (2011; Kordan riv.), Ghasemi and Kamali (2014; Haraz riv.), Egglishaw (1980; Jajrud, Bareghan riv.), Imanpour Namin et al. (2013; Tajan riv.), Shokri et al. (2014; Tajan riv.), Mousavi and Hakobyan (2017; Haraz riv., Dalir riv., Chatan riv., Firuz Abad riv.), Seyyedsharifi et al. (2014; Gharasou riv.), Farasat and Sharifi (2014; Kavat riv.), Mahboobi Soofiani et al. (2012; Zayanderud) and Braasch (1981, 50 km SE of Khorramabad).
Baetis (Baetis) baroukianus Thomas & Dia, 1984	Gilan Prov.: 7, 33, 34	Its distribution not known in details, reported from two disjunctive subareas in Lebanon and Iran (Thomas and Dia 1984, 1999, Godunko et al. 2017).	*B. alpinus* species-group. Described from Lebanon (Thomas and Dia 1984, 1999). First record in Iran by Godunko et al. (2017) was based on our material (loc. 7).
*Baetis (Baetis) bicaudatus Dodds, 1923	Tehran Prov.: Jajrud	Holarctic species, in Palaearctic Region reported from Altai, Mongolia and Russian Far East (Kluge 1997b).	A species close European representatives to the *B. alpinus* species-group. Reported by Amri et al. (2014). The occurrence in Iran is rather unlikely as its westernmost records were published from Mongolia.
**Baetis (Baetis) buceratus Eaton, 1870**	Gilan Prov.: 5, 13, 27, 28, 32, 37; Ardabil Prov.: 24, 38	Widely distributed from Europe to Central Asia including Near East, Iraq (Al-Zubaidi et al. 1987) and Turkey (Kazancı and Türkmen 2012, Salur et al. 2016).	Iran falls within its known distributional range.
**Baetis (Baetis) monnerati Gattolliat & Sartori, 2012**	Mazandaran Prov.: brook above Yalrud 36°06'17"N / 51°50'14"E, 2 larvae; brook above Molla Kala, 4 larvae (coll. M. Svitok, unpublished)	Recently described from Jordan (Gattolliat et al. 2012).	*B. buceratus* species-group. Iran represents the easternmost limit of its area.
**Baetis (Baetis) cf. nexus Navás, 1918**	Gilan Prov.: 14; Ardabil Prov.: 21–24, 26, 38	Known from Europe and Turkey (Kazancı 1985).	Iran represents easternmost limit of its area. Material shows some morphological differences from European material and requires more detailed examination. The synonymy of *B. pentaphlebodes* to *B. nexus* is highly questionable, we follow the IZCN Opinion No. 2171 (2007), until new evidence is published.
Baetis (Baetis) fuscatus (Linnaeus, 1761)	„Southern Persia“New recordsGilan Prov.: 1, 6, 13, 18, 27, 32, 36	Transpalaearctic species. Doubtfully distinguishable species from West Palaearctic species *B. scambus* Eaton, 1870 in the larval stage.	*B. fuscatus* mentioned by Eaton (1885) as “… eastwards to Southern Persia (Hagen Mus)”. Iran falls within known distributional range of both species.
**Baetis (Baetis) lutheri Müller-Liebenau, 1967**	Gilan Prov.: 1–7, 9, 11, 13, 14, 17, 18, 27, 28, 32, 35–37; Ardabil Prov.: 23, 24	Widely distributed from Europe to Caucasus, Turkey (Kazancı and Türkmen 2012, Salur et al. 2016) and Iraq (Al-Zubaidi et al. 1987).	Iran represents the easternmost limit of its area. Larvae of *B. lutheri* species-group from N Iran can be confused with poorly known species *Baetis petrovi* Tshernova, 1938 (see Soldán and Godunko 2008).
**Baetis (Baetis) vardarensis Ikonomov, 1962**	Gilan Prov.: 2, 5, 13, 14, 18,–28, 32, 36, 37; Ardabil Prov.: 26	Widely distributed from Europe to Caucasus and Turkey (Kazancı and Türkmen 2012, Salur et al. 2016).	Iran represents the easternmost limit of its area.
**Baetis (Baetis) samochai Koch, 1981**	Gilan Prov.: 13	Known from Turkey (Koch 1985), Israel, Lebanon and Syria (Koch 1981).	Iran represents the easternmost limit of its area.
Nigrobaetis (Takobia) muticus (Linnaeus, 1758)	Mazandaran Prov.: Chatan riv.New recordsGilan Prov.: 12, 19, 29, 35, 36; Ardabil Prov.: 21, 22	Widely distributed from North Africa (confirmation needed), Europe, Russia and Turkey to Caucasus and Central Asia (eastern Kazakhstan, Novikova and Kluge 1994). Known also from Korean peninsula and Japan.	In Iran, reported first from Chatan riv. in Mazandaran Prov. by Mousavi and Hakobyan (2017).
**Nigrobaetis (Nigrobaetis) gracilis (Bogoescu & Tabacaru, 1957)**	Gilan Prov.: 13	Distributed in the Alps, Carpathians, Caucasus, reported also from Tajikistan (Zimmermann 1981).	Iran falls within its known distributional range.
**Baetis (Rhodobaetis) braaschi (Zimmermann, 1980)**	Gilan Prov.: 9, 14; Ardabil Prov.: 22, 24–26	Occurs in neighbouring countries, reported from Eastern Ukraine to Crimea, Turkey, Caucasus and Central Asia (Sroka et al. 2012).	Iran represents the easternmost limit of its area.
**Baetis (Rhodobaetis) cf. vadimi Godunko, Palatov & Martynov, 2015**	Gilan Prov.: 7, 10–12, 14, 29, 31, 33; Ardabil Prov.: 19, 23	Probably undescribed species, closely related to *B. vadimi* from Georgia and Turkey. Possibly conspecific with part of material identified as “*Baetis gemellus*“ in the past from Europe and Middle East.	Material from Iran morphologically similar to species identified as B. cf. gadeai from Caucasus (Sroka 2012).
**Baetis (Rhodobaetis) ilex (Jacob & Zimmermann, 1978)**	Tehran Prov.: brook in Younza Pass, 35°59'18"N / 51°43'13"E, 5 larvae; brook 36°00'54"N /E 51°47'18‘‘, 7 male imagines (coll. M. Svitok, unpublished); Gilan Prov.: 12, 33; Ardabil Prov.: 19, 20, 22	Poorly known species, so far considered endemic to the Caucasus (Jacob and Zimmermann 1978).	Only 20 larvae known from the Caucasus to date (Jacob and Zimmermann 1978). Findings from Iran represent the second published records on its so far insufficiently known area.
Baetis (Rhodobaetis) rhodani (Pictet, 1843)	West Azerbaijan Prov.: Zarrinehrud; Alborz Prov.: Karaj riv.New recordsGilan Prov.: 1–7, 9–14, 27, 28, 30, 32–37; Ardabil Prov.: 16–23, 25, 26, 38	Widely distributed in the Western Palaearctic region. Records from the East Palaearctic appear rather unlikely (Bauenrfeind and Soldán 2012).	Reported from Zarrinehrud in West Azerbaijan Prov. (Ahmadi et al. 2012) and from Karaj riv. in Alborz Prov. (Khatami 2017). Iran falls within its known distributional range.
*Cloeon* sp.	Alborz Prov.: Kordan riv.; Mazandaran Prov.: Tajan riv., Valasht lake	Almost cosmopolitan, including some remote oceanic islands. About 15 species from three subgenera known from the West Palaearctic Region.	Unidentified species reported by Mousavi Nadushan and Ramezani (2011; Kordan riv.), Imanpour Namin et al. (2013; Tajan riv.) and Mousavi and Hakobyan (2017; Valasht lake).
Cloeon (Cloeon) cognatum Stephens, 1836	Tehran Prov.: Jajrud	Holarctic species, reported from Central America as well (McCafferty and Waltz 1990). The species requires the revision of the status.	Reported from Jajrud near Tehran (Amri et al. 2014).
Cloeon (Cloeon) dipterum (Linnaeus, 1761)	Tehran Prov.: JajrudNew recordArdabil Prov.: 38	Widely distributed in the Palaearctic Region, known also from the Nearctic Region (Quebec and Ontario, see Bauernfeind and Soldán 2012).	Except our record known from Jajrud near Tehran (Amri et al. 2014).
Cloeon (Similicloeon) simile Eaton, 1870	West Azerbaijan Prov.: Zarrinehrud	Transpalearctic species, missing in Japan.	Reported from Zarrinehrud in NW Iran (Ahmadi et al. 2012) and Jajrud near Tehran (Amri et al. 2014).
*Centroptilum* sp.	Isfahan Prov.: ZayanderudNew recordsGilan Prov.: 28, 31	Holarctic genus, with an area extension into the Oriental Region. Two West Palaearctic species: *C. luteolum* O. F. Müller, 1776 and *C. pirinense* Ikonomov, 1962.	Our records represent undescribed species related to *C. luteolum*. The species will be described by Martynov (pers. comm.) based on the material from Caucasus (AR Adjara). Unidentified species of the genus *Centroptilum* was also reported by Mahboobi Soofiani et al. (2012) from Zayanderud in Central Iran.
**Procloeon (Pseudocentroptilum) caspicum sp. n.**	Gilan Prov.: 7, 27 (type locality), 36	So far known from the type locality in Iran only.	
Oligoneuriidae			
*Oligoneuriella* sp.	Mazandaran Prov.: Tajan riv., Firuz Abad riv., Poleocean riv.; Isfahan Prov.: Zayanderud	Palaearctic genus, ten species known from the West Palaearctic Region. In the Near East, seven species known from Turkey (Kazancı and Türkmen 2012, Sroka et al. 2015), one from Iraq (Al-Zubaidi et al. 1987) and one from Syria (Koch 1980).	Unidentified species of the genus *Oligoneuriella* was reported from Zayanderud in Central Iran by Mahboobi Soofiani et al. (2012) and from Tajan riv., Firuz Abad riv. and Poleocean riv. in Mazandaran Prov. by Shokri et al. (2014) and Mousavi and Hakobyan (2017).
*Oligoneuriella tskhomelidzei* Sowa & Zosidze, 1973	Mazandaran Prov.: mountain stream, Gazanak, 1400 m a.s.l.New recordsGilan Prov.: 11, 17, 27, 36, 37	Caucasian species described from Georgia (Sowa and Zosidze 1973), known also from Turkey (Kazancı and Türkmen 2012, Salur et al. 2016).	*Oligoneuriella baskale* described from east Turkey, two female imagines reported from Iran (Soldán and Landa 1977). Later, the species was synonymised with *O. tskhomelidzei* by Kluge (2004), however without any supporting argumentation.
Heptageniidae			
*Arthroplea sp.	Isfahan Prov.: Zayanderud	Holarctic genus, in the Palaearctic Region evidently boreomontane element. One species, *Arthroplea congener* Bengtsson, 1908, in the West Palaearctic Region.	Unidentified species reported by Mahboobi Soofiani et al. (2012). The occurrence of this genus in Iran is highly unlikely as the most southern records of the genus were published from high altitudes in Switzerland, France and Ural Mts. (Bauernfeind and Soldán 2012).
*Ecdyonurus* sp.	Mazandaran Prov.: Tajan riv.; Qazvin Prov.: Shahrud; Alborz Prov.: Karaj riv.; Isfahan Prov.: ZayanderudNew recordsGilan Prov.: 14; Ardabil Prov.: 19–22, 25, 26	West Palaearctic genus, about 42 species known.	Unidentified species were reported by Shokri et al. (2014; Tajan riv.), Sharifinia et al. (2016a,b; Shahrud), Khatami (2017, Karaj riv.) and Mahboobi Soofiani et al. (2012; Zayanderud).Larvae and imagines related to *Ecdyonurus ornatipennis* from our material deserve further examination.
*Ecdyonurus ornatipennis* Tshernova, 1938	„117 km south of Rasht and 50 km SE of Khorramabad, 1500 m a.s.l.“	Described from Azerbaijan, known throughout Caucasus and from Turkey (Kazancı and Türkmen 2012, Salur et al. 2016).	First records from Iran by Braasch (1981) with insufficient localisation. Recently reported from Talysh Mts. close to Iranian border by Palatov and Sokolova (2006).
*Electrogena bothmeri* (Braasch, 1983)	Chalus, Mazandaran Prov. (type locality)	Known only as the holotype male subimago (!) described by Braasch (1983a).	Single record from the type locality (Braasch 1983a), no record since then.
***Electrogena pseudaffinis* (Braasch, 1980)**	Gilan Prov.: 1–4, 6, 7, 10–12, 15–18, 27, 28, 30–32, 35, 36	Caucasian species described from the Russian part of Caucasus (Braasch 1980a). Known from Russia and Georgia (Braasch 1980a,b, Martynov et al. 2016), Turkey (Kazancı and Braasch 1988, Kazancı and Türkmen 2012, Salur et al. 2016) and Azerbaijan (coll. Soldan, unpublished).	Common and often abundant species in streams studied in the Gilan Prov., preferring forest streams and rivers at lower altitudes.
**Electrogena cf. squamata (Braasch, 1978)**	Gilan Prov.: 10–12, 16, 28, 29, 31–35	Caucasian species known from Georgia (Braasch 1978, 1980b, Martynov et al. 2016), Russia (Braasch 1978b) and Azerbaijan (Braasch 1980b).	Common and often abundant species in streams studied in Gilan Prov., preferring forest streams and rivers with no apparent altitude preference. At lower stream sections syntopic with *E. pseudaffinis*.
*Electrogena ressli* (Braasch, 1981)	Gilan Prov.: Rasht	Type locality in Turkey, paratypes (one male imago and one male subimago) known from Iran (Braasch 1981).	Single record from the type locality, no record since then.
*Heptagenia* sp.	Tehran Prov.: Jajrud, Bareghan riv.; Alborz Prov.: Karaj riv.; Mazandaran Prov.: Haraz riv., Tajan riv.; Isfahan Prov.: Zayanderud	Holarctic and Oriental genus, not recorded from North Africa. Nine species known from the West Palaearctic Region. Five species known from the Near East.	Unidentified species reported by Egglishaw (1980; Jajrud, Bareghan riv.), Shayeghi et al. (2015; Karaj riv.), Ghasemi and Kamali (2014; Haraz riv.), Shokri et al. 2014; Tajan riv.), and Mahboobi Soofiani et al. (2012; Zayanderud).
*Heptagenia samochai* Demoulin, 1973	Golestan Prov.: Gorgan	Known from eastern Europe to Asia Minor. Recorded from Georgia, Crimean Peninsula, Russia, Armenia, Israel, and Iran.	Reported from Iran sub *Heptagenia lutea* (syn. subj.) by Kluge (1987).
*Epeorus* sp.	Alborz Prov.: Kordan riv.; Mazandaran Prov.: Tajan riv.; Ardabil Prov.: Gharasou riv.	Holarctic genus, with an extension to Neotropics and Oriental Region. Representatives of three subgenera, *Caucasiron*, *Epeorus* and *Ironopsis*, (Kluge 1997a) known from the West Palaearctic Region.	Unidentified species without an affiliance to either subgenera were reported by Mousavi Nadushan and Ramezani (2011; Kordan riv.), Imanpour Namin et al. (2013; Tajan riv.), Shokri et al. (2014; Tajan riv.) and Seyyedsharifi et al. (2014; Gharasou riv.).
* Epeorus (Iron) sp.	Tehran Prov.: Jajrud, Bareghan riv.	Subgenus Iron is Holarctic, its species known mainly from Central Asia, Siberia, Far East and North America (Kluge 1997b, Kluge 2004).	Unidentified species reported by Egglishaw (1980) from Jajrud and Bareghan rivers likely refer to some species of *Epeorus* known from the north Iran.
* Epeorus (Ironopsis) sp.	Tehran Prov.: Jajrud	Subgenus Ironopsis is Holarctic, its species known from USA, Central Asia and Europe (Kluge 1997b, Kluge 2004).	Unidentified species reported by Egglishaw (1980) from Jajrud likely refer to some species of *Epeorus* known from the north Iran.
Epeorus (Caucasiron) sp.	Gilan Prov.: 12, 17, 27, 30	Subgenus Caucasiron is distributed in the East Mediterranean, Caucasus, Central Asia and Southwestern China. Eleven species and two subspecies known up to date. The highest diversity (9 species) in the Caucasus Mts.	Species recorded in low abundace in Gilan Prov.; deserves further examination.
Epeorus (Caucasiron) caucasicus iranicus (Braasch & Soldán, 1979), comb. nov.	Tehran Prov.: stream in Darband Valley, 2100 m a.s.l., (type locality).Mazandaran Prov.: Dalir riv., Firuz Abad riv., Haraz riv., Koshk Sara riv.New recordArdabil Prov.: 19	Recently known only from the Alborz mountain range. Larva and nymphal protopenis bear features proposed for subgenus Caucasiron, imago unknown.	Recorded from Tehran Prov. (Braasch and Soldán 1979), Mazandaran Prov. (Mousavi and Hakobyan 2017), and in several individuals also from the Ardabil Prov.
**Epeorus (Caucasiron) cf. znojkoi Tshernova, 1938**	Gilan Prov.: 2–4, 7, 8, 10–12, 15–17, 27, 29, 30, 33–35	Widely distributed in Caucasus and Asia Minor. Known from Turkey (Türkmen and Kazancı 2015, Salur et al. 2016), Georgia (e.g., Martynov et al. 2016), Armenia (Sinitshenkova 1976), Russia (e.g., Chen 1999) and Azerbaijan (e.g., Sinitshenkova 1976).	The most common Epeorus (Caucasiron) species recorded at the streams studied in Gilan Prov. Iran represents the easternmost limit of its known distribution.
**Epeorus (Epeorus) zaitzevi Tshernova, 1981**	Gilan Prov.: 14	Described from Armenia as imago, larva described by Demoulin (1973) as *Epeorus* sp. and Braasch (1978a) as *Epeorus znojkoi*. Widely distributed in Caucasus and Near East: Turkey (Kazancı and Türkmen 2012, Salur et al. 2016), Israel (Sartori 1992), Iraq (Al-Zubaidi et al. 1987), Syria (Koch 1988), Azerbaijan (Chen 1999) and Georgia (coll. Hrivniak, unpublished).	Species recorded from one locality in the Alborz Mts. Iran represents the easternmost limit of its known distribution.
*Cinygmula sp.	Qazvin Prov.: Shahrud	*Cinygmula* shows Holarctic (East Palaearctic and Nearctic) area. Western limits of this area in Central Asia (Uzbekistan, Kirgizstan) and probably northern mountain ranges in Afghanistan and Pakistan, definitively missing in Caucasus.	Unidentified species reported by Sharifinia et al. (2016a,b). Most probably misidentification at the generic level (*Rhithrogena*?), the occurrence of any representative of *Cinygmula* in Iran very unlikely.
*Rhithrogena* sp.	Tehran Prov.: Jajrud, Bareghan riv.; Alborz Prov.: Kordan Riv.; Mazandaran Prov.: Tajan Riv.; Isfahan Prov.: Zayanderud;New recordGilan Prov.: 19	Holarctic genus, including North Africa, with the area extension to the Oriental Region. Very diverse genus (more than 150 species) in the West Palaearctic Region.	Unidentified species reported by Egglishaw (1980; Jajrud, Bareghan riv.), Mousavi Nadushan and Ramezani (2011; Kordan riv.), Shokri et al. (2014; Tajan riv.) and Mahboobi Soofiani et al. (2012; Zayanderud).
**Rhithrogena cf. decolorata Sinitshenkova, 1973**	Gilan Prov.: 10–12, 15, 17, 18, 27, 33, 34, 35–37	Widely distributed throughout the Caucasus, known also from the Talysh Mts. in Azerbaijan (Palatov and Sokolova 2016).	Common species in the Gilan Prov.
*Rhithrogena iranica* Braasch, 1983	Shesavar (type locality), likely referring to Shahsavar	Known only as the holotype (male imago) and paratypes (two female subimagines) described by Braasch (1983b) from a single locality.	Insufficient localisation of the type locality.
*Rhithrogena paulinae* Sartori & Sowa, 1992	Tehran Prov.: Sefid Khok, Alborz Mts., 2200 m a.s.l. (type locality)	Only holotype (imago male) and paratypes (four female imagos and two larvae) from a single locality known (Sartori and Sowa 1992).	Known only from the Alborz Mts.
Leptophlebiidae			
*Paraleptophlebia* sp.	Alborz Prov.: Kordan riv.	Holarctic genus, six species known from the West Palaearctic Region.	Unidentified species reported by Mousavi Nadushan and Ramezani (2011) from Kordan riv. in Alborz Prov.
*Paraleptophlebia submarginata* (Stephens, 1935)	„50 km SE of Khorramabad, 1500 m a.s.l.“	Widely distributed in Europe (from Fennoscandia to Mediterranean), in northeast reaching to Ural and W Siberia (e.g., Novikova 1984, Beketov and Kluge 2003), and southeast to Israel (Sartori 1992) and Iran (Braasch 1981).	The only record from Iran with insufficient localisation (Braasch 1981).
***Habroleptoides confusa* Sartori & Jacob, 1986**	Gilan Prov.: 7, 8, 10, 12, 15, 16, 27–29, 31, 33–35.	Widely distributed in Europe (not in Fennoscandia), in east from Greece and Turkey to Armenia and Azerbaijan (Sartori and Jacob 1986). Iran represents the easternmost limit of its area.	Common in small forest brooks in the Gilan Province.
**Habrophlebia cf. lauta Eaton, 1884**	Gilan Prov.: 1, 8, 31	West Palaearctic species, known from North Africa, Europe, Caucasus and Turkey.	Only small-instar larvae found in the Gilan Prov.
*Leptophlebia sp.	Mazandaran Prov.: Tajan riv.	Holarctic genus, with extension to transitory Palaearctic-Oriental area in China. Only two West-Palaearctic species, *L. vespertina* Linnaeus, 1758 and *L. marginata* Linné, 1767, which occurrence in Iran is unlikely.	Unidentified species reported by (Imanpour Namin et al. 2013).
Choroterpes (Euthraulus) sumbarensis Kluge, 1984	Razavi Khorasan Prov.: Mashhad (Kopedag Mts.)	Described from the Kopetdag Mts. in Turkmenistan (Kluge 1984).	According to Mohammadian (2005), Kluge (pers. comm.) reported the species from Mashhad.
Ephemerellidae			
*Ephemerella* sp.	Mazandaran Prov.: Tajan riv.; Alborz Prov.: Karaj riv.	Holarctic and Oriental genus, three species known from the West Palaearctic Region.	Unidentified species reported by Khatami (2017) from Karaj riv. and by Shokri et al. (2014) and Imanpour Namin et al. (2013) from Tajan riv. in Mazandaran Province.Specimens from our collection in the Ardabil Prov. require further examination.
*Ephemerella maculocaudata Ikonomov, 1961	Mazandaran Prov.: Siah Bisheh riv.	Mediterranean species known from two disjunctive areas, Balkan (Macedonia, Bulgaria) and west Mediterranean (Spain, France). Occurrence in Iran is unlikely.According to Jacobus and McCafferty (2008) the species was synonymised with *Teloganopsis mesoleuca* (Brauer, 1857) which was recently not confirmed by Bauernfeind and Soldán (2012).	Recorded from Iran as *Ephemerella maculocaudata* Ikonomov, 1961 by Mousavi and Hakobyan (2017).
*Serratella* sp.	Mazandaran Prov.: Shahrud; Isfahan Prov.: Zayanderud	Holarctic and Oriental Regions. Generic classification of species is unstable in the literature.	Unidentified species reported by Sharifinia et al. (2016a,b) from Shahrud and Mahboobi Soofiani et al. (2015) from Zayandehrud.
*Serratella elissa* Jacobus, Zhou & McCafferty, 2009	Gilan Prov.: Gilan River (?) at Lanak Waterfall, 37°00'N, 49°52'E (type locality); Havigh River, 20 km south of AstaraNew records1–4, 6, 7, 11, 15–18, 27, 28, 32, 35, 36	Described from the Gilan Province by Jacobus et al. (2009).	Common species at our streams studied; can occur at high abundance in eutrophicated streams. Found also at the type locality in Lunak (not Lanak in page 55 in Jacobus et al. 2009) waterfall.
*Serratella ignita* (Poda, 1761)	West Azerbaijan Prov.: ZarrinehrudNew recordsGilan Prov.: 1–4, 6, 18; Ardabil Prov.: 21, 25	Widely distributed species, known from North Africa and entire Europe, through Asia Minor, Near East to Mongolia, China and Korea.	Reported from Zarrinehrud in NW Iran (Ahmadi et al. 2011, 2012). In our material, not as frequent and abundant as *S. elissa* at studied streams.
*Teloganopsis subsolana* (Allen, 1973)	Mazandaran Prov.: 13 km NW of Ghalekesh	Described from the Kabul River in Afghanistan (Allen 1973).	The only record since its original description(Jacobus 2009).
Potamanthidae			
*Potamanthus* sp.	Isfahan Prov.: Zayanderud	Holarctic and Oriental genus, including single Palearctic species *Potamanthus luteus* Linné, 1767. Two subspecies currently recognised: *P. luteus luteus* Linné, 1767 and *P. luteus oriens* Bae & McCafferty, 1991. The former distributed in Europe, North Africa and Asia Minor (Turkey and Syria) and the latter distributed from lower Amur basin to Manchuria, Japan and Korea.	Unidentified species of the genus *Potamanthus* reported by Mahboobi Soofiani et al. (2012) from Zayanderud. Family Potamanthidae was reported by Nemati Varnosfaderany et al. (2010) from the same river.
Ephemeridae			
*Ephemera danica* (Müller, 1764)	West Azerbaijan Prov.: Zarrinehrud	West Palaearctic species, distributed in Europe and southeast to Turkey (Kazancı and Türkmen 2012, Salur et al. 2016) and Liban (Thomas et al. 2007).	Reported from Zarrineh river in W Azerbaijan (Ahmadi et al. 2012).
Palingeniidae			
*Mortogenesia mesopotamica* (Morton, 1921)	Karkheh riv., Bsaitin (?)	Described and later confirmed by several records from Tigris river in Iraq (see references in Soldán and Godunko 2013).	Soldán and Godunko (2013) studied the material from Iran (Karkheh riv.). However, proper locality cannot be identified.
*Palingenia fuliginosa* (Georgi, 1802)	Gilan Prov.: Hassankiade	Known from eastern Europe (E Slovakia, N Ukraine), Caucasus Mts., and northern Iran.	The only historical record by Tshernova (1949) probably refers to the village Hasan Kiadeh on Sefid Rud river.
*Palingenia longicauda* (Olivier, 1791)	Aras riv.	South-Central European species.	The record is based on 5 male imagines collected on 20 June 1905 available in the collection of the Museum für Naturkunde in Berlin. Material was revised by A.H. Staniczek and R.J. Godunko in February 2017. Previous determination “Palingenia longicauda Oliv var. fuliginosa Georgi“ by E. Schoenemund. As Aras river forms the border between Azerbaijan and Iran, the species can be formally included in the Iranian fauna.
*Palingenia orientalis* Chopra, 1927	Sistan and Baluchestan Prov.: Seistan (?) (type locality)	Known from two discrete areas; described from “Seistan” by Chopra (1927) and later recorded from Israel (Sartori 1992).	The record from Iran is based on the type series only (Chopra 1927) which is, however, insufficiently localised (“Seistan, Persia”). The author described the species based on imagines and mentioned that “the nymphs have been described by Graverly in detail”. Graverly (1920) described the nymphs as “Palingenia (s. str.) ? longicauda, Olivier“ and the material is localised as „Randa stream 4 miles NW of Jellalabad, Seistan“.
Caenidae			
*Caenis* sp.	Tehran Prov.: Jajrud, Bareghan riv.; Alborz Prov.: Kordan riv.; Mazandaran Prov.: Tajan riv., Haraz riv.; Isfahan Prov. Zayanderud	Almost cosmopolitan genus, except for Australia and remote oceanic islands. At least 22 species known from the West Palaearctic Region.	Unidentified species reported by Egglishaw (1980; Jajrud, Bareghan riv.), Mousavi Nadushan and Ramezani (2011; Kordan riv.), Imanpour Namin et al. (2013; Tajan riv.), Shokri et al. (2014; Tajan riv.), Ghasemi and Kamali (2014; Haraz riv.) and Mahboobi Soofiani et al. (2012; Zayanderud).
*Caenis kopetdagi* Kluge, 1985	Razavi Khorasan Prov.: Mashhad (Kopedag Mts.)	Described from the Kopetdag Mts. in Turkmenistan (Kluge 1985).	According to Mohammadian (2005), Kluge (pers. comm.) reported the species from Mashhad.
*Caenis macrura* Stephens, 1836	Mazandaran Prov.: Koshk Sara riv., Abbas Abad Dam, Valasht lakeNew recordsGilan Prov.: 1–4, 6, 8–11, 14–18, 27, 30–32, 35–37; Ardabil Prov.: 20–22, 24–26, 38	Palaearctic species distributed from Fennoscandia east to Russia and Minor Asia. Known from Israel (Malzacher 1992), Syria (Koch 1988) and Iraq (Al-Zubaidi et al. 1987).	Records from Mazandaran Prov. provided by Mousavi and Hakobyan (2017).
*Cercobrachys* sp.	Isfahan Prov.: Zayanderud	Holarctic, Oriental and Neotropic genus. Single Palaearctic species, *C. minutus* Tshernova, 1952 with wide Transpalaearctic distribution.	Unidentified species reported by Mahboobi Soofiani et al. (2012)
*Clypeocaenis bisetosa* Soldán, 1978	Mazandaran Prov.: mountain stream in Gazenak, 1400 m a.s.l.	Described from India, paratypes from the Alborz Mts. (Soldán 1978a).	No recent record from Iran.

^#^ Nearctic/Neotropic species and genera reported from Iran, which are definitely misidentifications:Jajrud near Tehran (Amri et al. 2014): *Baetis adonis*, *B. bicaudatus*, *B. alius*, *B. magnus*, *B. notos*, *B. persecutor*, *B. tricaudatus*, *Epeorus albertae*, *E. fragilis*, *E. hesperus*, *E. grandis*, *Rhithrogena exilis*, *R. ingalik*, *Caenis tardata*, *Paraleptophlebia adoptiva*, *P. clara*, *P. debilis*, *Lachlania fusca*, *L. lucida*, *L. iops*. Karaj riv., Alborz Prov. (Shayeghi et al. 2015): *Maccaffertium* sp. Zarrinehrud, West Azerbaijan Prov. (Ahmadi et al. 2011, 2012): *Callibaetis nigritus*, *Campsurus notatus*. Kavat riv., Kermanshah Prov. (Farasat and Sharifi 2014): *Ephemerella doris*, *Maccaffertium* sp. Zayanderud, Isfahan Prov. (Mahboobi Soofiani et al. 2012): *Attenela* sp., *Heterocloeon* sp. Shapour riv., Fars Prov. (Golchin Manshadi and Ghafari 2015): *Tricorythodes* sp.

Mayfly larvae were collected by T. Soldán, J. Bojková and J. Imanpour Namin from 12 to 22 May 2016, using metal strainers after kick-sampling. Sampling of larvae for about 30–60 minutes was supplemented by sweeping of imagines and subimagines from riparian vegetation by a standard entomological net. The material studied in the present contribution sums up to 9213 larval specimens and 245 subimagines and imagines. Most material is deposited in the collection of the Biology Centre, Czech Academy of Sciences, Institute of Entomology, České Budějovice, Czech Republic. Reference specimens for the species recorded are deposited in the collection of J. Imanpour Namin (Department of Fishery, University of Gilan). All specimens were preserved in 96% ethanol. Some specimens were mounted on slides with HydroMatrix (MicroTech Lab, Graz, Austria). Drawings for the descriptions of the new species were made using a stereomicroscope Olympus SZX7 and a microscope Olympus BX41, both equipped with a drawing tube. Photographs were made using a Canon EOS 1200D camera mounted on a Leica M205 C stereomicroscope. All photographs were subsequently enhanced with Adobe Photoshop CS5. For scanning electron microscopy, samples were gradually transferred to acetone, critical point dried and coated with gold by sputtering using a Baltec SCD050 Sputter Coater. Observations were taken on the scanning microscope Jeol JSM 7401F at 4 kV (BC CAS, České Budějovice). Eggs were dissected from a pharate female subimago.

## Results and discussion

### Procloeon (Pseudocentroptilum) caspicum

Taxon classificationAnimaliaEphemeropteraBaetidae

Sroka sp. n.

http://zoobank.org/23B02170-B45C-4782-8473-8E20280EA31C

#### Diagnosis

(based on larvae and eggs). Labrum with pronounced medial notch, anterior margin laterally from medial notch strongly asymmetric; mandible incisor groups separated at distal third of their length; maxillary palps three-segmented, not thickened, length of segment III of maxillary palp reaches 0.5 × segment II length; fully developed hind wing pads; length of tarsal claws 0.44 × tarsus (forelegs); 0.55 × tarsus (middle and hind legs); lateral spines present on abdominal segments VIII–IX; single gill plates with rudimental dorsal lamella; inner margin of paraproct with approximately 8–11 large teeth; egg chorion without equatorial band of large papillae.

#### Description.

**Figures 2–11.**
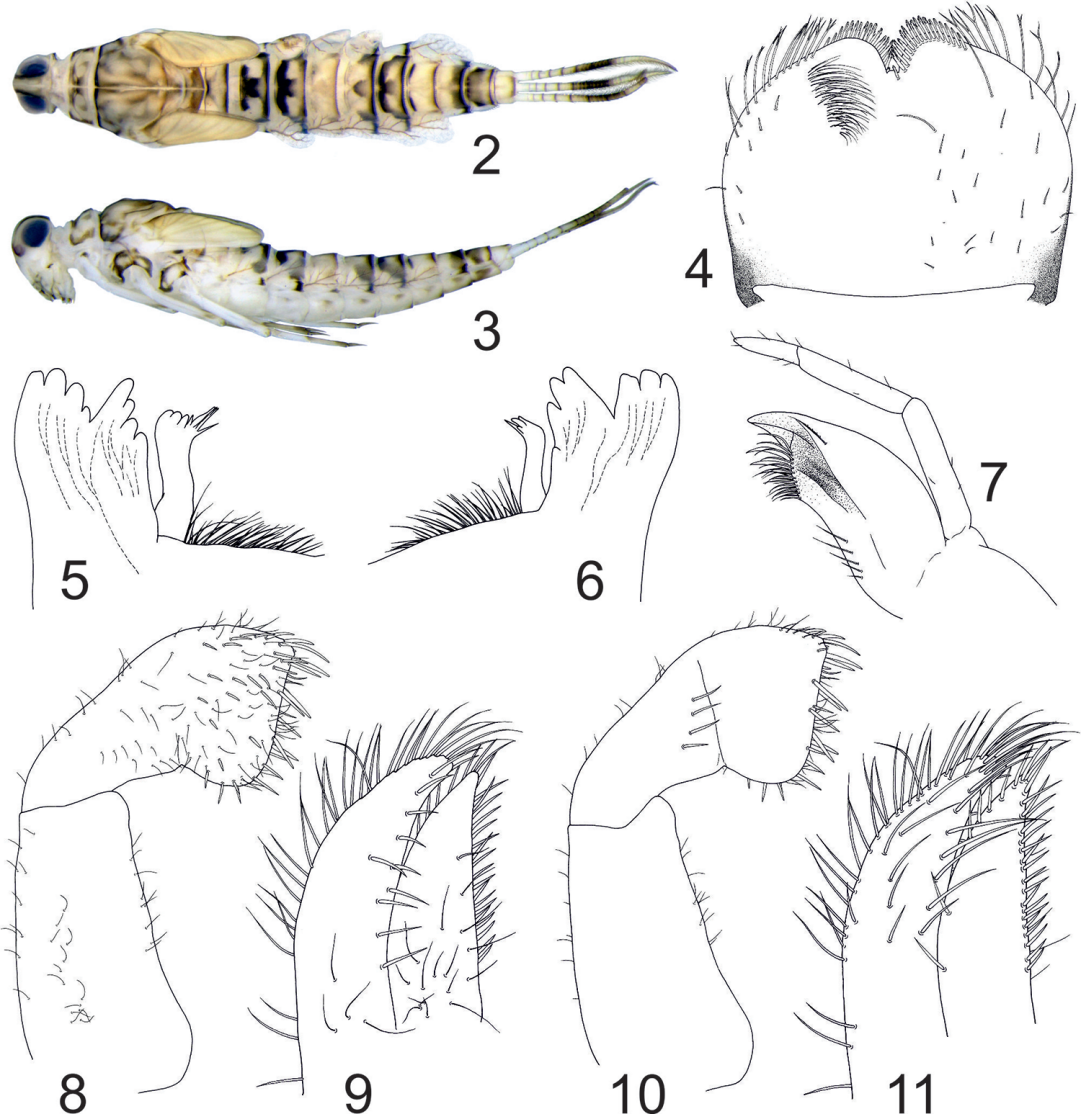
Procloeon (Pseudocentroptilum) caspicum sp. n., larva: **2** mature female larva, habitus (dorsal) **3** mature female larva, habitus (lateral) **4** labrum (right side dorsal, left side ventral) **5** left mandible, apicolateral part (dorsal) **6** right mandible, apicolateral part (dorsal) **7** maxilla **8** labial palp (ventral) **9** glossa and paraglossa (ventral) **10** labial palp (dorsal) **11** glossa and paraglossa (dorsal).

***Mature larva.*** Body length 7–8 mm, length of antennae approximately 2 mm, length of cerci 2–3 mm (0.3 × body length). General colouration yellowish with darker brownish pattern (Figs 2, 3).

*Head.* Labrum (Fig. 4) approximately 1.3 × broader than long, anterior margin with pronounced medial notch. Anterior margin laterally from medial notch strongly asymmetrically rounded. Along anterior margin, row of bifurcated setae situated anterolaterally and shorter, stout setae anteromedially. Dorsal surface with scattered hair-like setae, not arranged in rows; ventral surface with group of hair-like setae medially. Mandible incisors (Figs 5, 6) divided into two groups, separated at distal third of their length. Each group with 3–4 rounded denticles. Left prostheca broadened apically, with approximately three blunt teeth and four longer sharp teeth (Fig. 5); right prostheca not broadened apically, with approximately three blunt teeth and one longer sharp tooth (Fig. 6). Group of long setae present between incisors and molar area. Maxillary palps (Fig. 7) 3-segmented, second slightly shorter than first segment. Third segment approximately half as long as second segment, apically tapering, bluntly pointed, without scales. Maxillary palps sparsely covered with tiny hair-like setae. Labial palps 3-segmented (Figs 8, 10), third segment with rounded angles and straight margin apically. Ventral side of first and second segment with scattered hair-like setae, third segment with several longer and thicker setae (particularly along apical margin) and numerous hair-like setae (Fig. 8). Dorsal side of second segment with group of 4–7 long setae, otherwise dorsal side of all segments without setae (Fig. 10). Glossae as broad as paraglossae, paraglossae slightly longer. Paraglossae ventrally with single irregular submarginal row of setae along inner margin, basal parts of glossae and paraglossae with sparse groups of long hair-like setae (Fig. 9). Glossae and paraglossae dorsally with rows of setae along margins (longer setae on paraglossae, shorter on glossae), denser setation apically, one additional irregular row of long setae in median portion of paraglossae (Fig. 11).

*Thorax*. Prothorax approximately 3× broader than long, whitish, with darker brownish pattern (Fig. 2). Mesothorax of same colour, metathorax darker posteriorly. Hind wing pads fully developed (Fig. 16). Legs pale yellowish, femora with darker brown smudges distally. Tibiae darker in proximal portion, tarsi darkened proximally and distally. Femora with oblique transversal row of hair-like setae subapically, extending to outer margin (Fig. 15). Curved row of hair-like setae proximal to tibio-patellar suture along outer margin of tibia (Fig. 15). Tarsi with sparse row of hair-like setae along outer margin in basal half of tarsus. Claws brownish, with numerous minute teeth arranged in two parallel rows, reaching approximately 2/5 of claw length (Fig. 29a, b). Measurements of individual leg segments (femur : tibia : tarsus : claw): 1.15 : 0.68 : 0.71 : 0.27 mm in foreleg, 1.18 : 0.67 : 0.65 : 0.29 mm in middle leg, 1.18 : 0.66 : 0.64 : 0.29 mm in hind leg (averages from six individuals).

**Figures 12–28.**
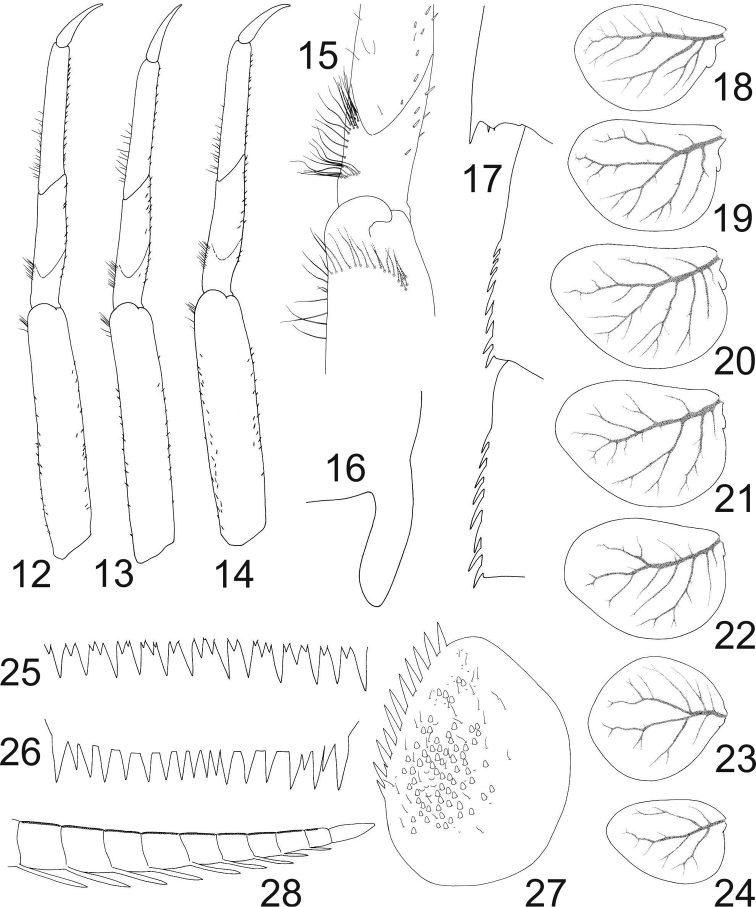
Procloeon (Pseudocentroptilum) caspicum sp. n., larva: **12** foreleg **13** middle leg **14** hind leg **15** foreleg, apical part of femur and basal part of tibia (dorsal, setae on ventral side dashed) **16** hind wing pad **17** lateral margin of abdominal segments VII–IX **18–24** gill plates I–VII **25** tergite V, posterior margin **26** tergite X, posterior margin **27** paraproct **28** cercus, apical part (swimming setae omitted).

*Abdomen.* Terga whitish, with dark spots forming clear pattern (Figs 2, 3). Terga I–VIII with dark spot posterolaterally (on segments I–VII near respective gill insertion). Tergum I with dark stripe on posterior margin. Tergum II with distinct dark patch medially, wide band (sometimes interrupted in middle) along anterior margin and thinner stripe on posterior margin, fused with enlarged smudges situated posterolaterally. Tergum III similar to tergum II, band along anterior margin more distinct, sometimes fused with posterolateral smudges. Tergum IV pale, with thin stripe on posterior margin and indistinct smudges medially and laterally. Terga V–VI with dark patch medially and dark stripe on posterior margin, fused with enlarged smudges situated posterolaterally. Tergum V also bears distinct dark band along anterior margin, connected to posterolateral smudges. Tergum VII with thin dark stripe on posterior margin and slightly wider stripe along anterior margin, interrupted in middle. Tergum VIII with wide dark band along posterior margin. Tergum IX with thin dark stripe on posterior margin and wide dark band anteriorly (most dark areas on anterior margin and laterally). Tergum X with dark stripe on posterior margin. Sterna pale whitish with dark patches sublaterally and dark stripe on posterior margin. Distinctiveness of this pattern increasing in more posterior segments. Sterna VIII–IX all dark smudged. Surface of abdomen covered with numerous scales and scale bases (Fig. 30); similar scales also on legs and other body parts. Posterior margin of abdominal terga I–IX with large teeth accompanied by smaller ones (Figs 25, 30). Teeth on tergum X smallest medially, lateral teeth slightly longer (Fig. 26). Segments II–VII with 1–2 prominent posterolateral spines near gill bases, sometimes accompanied by few smaller ones. Lateral spines present on segments VIII–IX (Fig. 17). Gills (Figs 18–24) whitish, with distinct tracheization. All gills simple, vestigial dorsal lamella present. Gills asymmetric and apically rounded. Paraproct (Fig. 27) with approximately 8–11 large teeth accompanied with scarce smaller ones on inner margin. Ventral surface of paraproct plate covered with scales, scale bases, and tiny hair-like setae. Caudal filaments reaching approximately 1/3 of body length, yellowish, with dark brownish stripe in middle. Paracercus slightly shorter than cerci. Ring of small triangular spines at each articulation of caudal filaments, alternated with larger spines every fourth segment (Fig. 31, these larger spines accompanied by dark brown stripe and distinction more pronounced in basal part of filament). Articulations further equipped with flattened scales and scale bases. Long swimming setae along inner margin of cerci and on both margins of paracercus. In basal third of filaments swimming setae only scarce, apically only last one or two segments without setae. Outer margin of cerci with enlarged, long, and thick spines on distal segments, longer than corresponding segment (Fig. 28).

**Figures 29–32.**
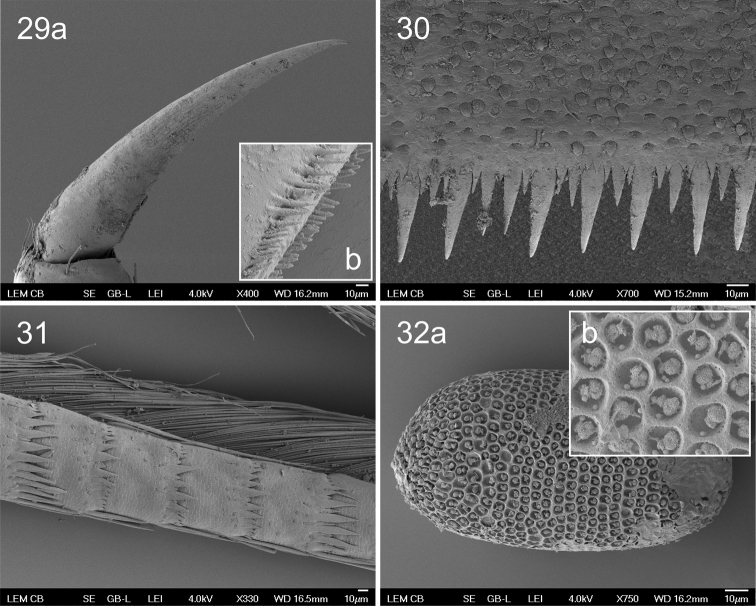
Procloeon (Pseudocentroptilum) caspicum sp. n., larva, egg: **29a** claw **29b** detail of claw teeth **30** tergite V surface and posterior margin **31** cercus, distal part **32a** egg **32b** detail of chorionic surface.

#### Egg.

Oval shaped; 130–140 µm long, 65–75 µm wide. Chorionic surface covered with thick reticulated ridges forming irregular polygonal mesh (Fig. 32a, b). Each polygon with coiled knob-like thread.

#### Imago and subimago.

Unknown.

#### Type material.

*Holotype*. Female mature larva, IRAN, Chelvand River above Chelvand (S of Lavandvil), approximately 2.5 km from its inflow into Caspian Sea, -6 m a.s.l., 38°17'20"N, 48°51'35"E (locality 27), 19.5.2016.

*Paratypes*. 1 male, 7 female larvae (3 specimens mounted on slides), same locality as holotype; 1 female larva, IRAN, Sefidab River in Divresh (SE of Shirkooh), 273 m a.s.l., 36°53'59"N, 49°35'06"E (locality 7), 13.5.2016; 1 female larva, IRAN, Karganrud River in Talesh, ca 7 km from its inflow into the Caspian Sea, 36 m a.s.l., 37°48'22"N, 48°54'27"E (locality 36), 22.5.2016.

All types deposited in the collection of the Biology Centre, Czech Academy of Sciences, Institute of Entomology, České Budějovice, Czech Republic.

#### Etymology.

The species name refers to the proximity of the type locality to the Caspian Sea.

#### Habitat.

Larvae were found in three different stream habitats, two eutrophic streams of different size (Chelvand and Karganrud rivers) in the Caspian Sea coastal plain relatively close to their inflow to the sea and one small, clear and cold brook in the forest. Chelvand at the type locality is a small river with coarse stony substratum rapidly flowing from the hills to the plain so it partially keeps its mountainous character also in low altitude (-6 m a.s.l.). *Procloeon caspicum* sp. n. larvae co-occurred with numerous larvae of *Serratella elissa*, *Baetis vardarensis* and *Caenis macrura*, and less numerous Epeorus (Caucasiron) spp., Rhithrogena cf. decolorata. Karganrud in Talesh is a warm river flowing in the urban and agricultural area with wide flat alluvium and stony-gravel substratum. Bottom substrate had rich cover of green filamentous algae. *Procloeon caspicum* sp. n. co-occurred with numerous larvae of Rhithrogena cf. decolorata, *Oligoneuriella tskhomelidzei*, *Baetis vardarensis*, *Baetis rhodani*, and less numerous *Baetis fuscatus* and *Serratella elissa*. Sefidab, the third and completely different stream, is a small cold brook entirely shaded by forest with coarse stony bottom and alternating pools and riffles. *Procloeon caspicum* co-occurred with numerous larvae of Epeorus (Caucasiron) cf. znojkoi, *Electrogena pseudaffinis*, *Baetis baroukianus* and less numerous *Serratella elissa* and *Habroleptoides confusa*.

## Affinities of Procloeon (Pseudocentroptilum) caspicum sp. n.

Within the subfamily Cloeoninae, several views on the (sub)generic classification have been published, most recently by Jacob (1991), Kluge and Novikova (1992), Bauernfeind and Soldán (2012), and Kluge (2016). All these authors recognize basically the same higher taxa, the difference is mostly in the hierarchical structuring and grouping of individual (sub)genera. All concepts use some characters of unclear polarity and/or derived characters prone to convergence to define individual taxa, thus all represent more or less “working versions” until a large-scale phylogenetic analysis of Baetidae is accomplished.

In this study, we follow Bauernfeind and Soldán (2012), where the new species is attributable to the genus *Procloeon* Bengtsson 1915. *Procloeon caspicum* sp. n. corresponds with all diagnostic characters given for *Procloeon* by Bauernfeind and Soldán (2012), most importantly the presence of long, blade-shaped, apicolateral spines in distal part of cerci. This character is suggested as synapomorphy of the clade *Procloeon* + *Pseudocentroptiloides* by Kluge and Novikova (1992), who treated both taxa as subgenera of *Cloeon*. Within *Procloeon* sensu Bauernfeind and Soldán (2012), the new species is attributable to the subgenus Pseudocentroptilum Bogoescu, 1947, based on the presence of hind wings and mandibular incisor groups separated in apical part only. This subgenus contains 18 species distributed in the Holarctic and Oriental regions (Bauernfeind and Soldán 2012).

The new species is characterized by a relatively uncommon (within *Procloeon*) combination of two characters, i.e., the presence of fully developed hind wing pads and single gill plates. Such a combination is present in three *Procloeon* species only, namely Procloeon (Pseudocentroptilum) albisternum (Novikova, 1986), Procloeon (Pseudocentroptilum) maritimum (Kluge, 1983) and Procloeon (Pseudocentroptilum) calabrum (Belfiore & D’Antonio, 1990). Occurrence of these species in Iran is extremely unlikely, since the former two species are distributed in the Far East – Russia (Novikova 1987, Kluge 1983, Tiunova 2009) and South Korea (Bae and Park 1997), and the latter species is endemic to a very small area of southern Apennine (Belfiore pers. comm.). Furthermore, these species can be differentiated from *P. caspicum* sp. n. using several morphological characters:

Both *P. albisternum* and *P. maritimum* differ from *P. caspicum* sp. n. in the absence of rudimental dorsal lamella of gill plates (figs 105–109 in Kluge 1983, fig. 2 in Novikova 1987). The extent of the dorsal lamella reduction may exhibit intraspecific variability within Cloeoninae (e.g., in related Procloeon (Pseudocentroptilum) heterophyllum Kluge & Novikova, 1992, the minute dorsal lamella may be present or absent, see figs 1–14 in Kluge and Novikova 1992).

However, in contrast to *P. caspicum* sp. n., *P. albisternum* possesses a different shape of labrum (almost rectangular with a very shallow notch in the middle of anterior margin), more deeply divided mandibular incisors, and a two-segmented maxillary palp (fig. 1 in Novikova 1987). *P. albisternum* is equipped with lateral spines on abdominal segments II–IX (only on segments VIII–IX in *P. caspicum* sp. n.) and has a different shape of gill plates, in particular gills II–IV being more asymmetric with the inner margin extended anteriorly (fig. 2 in Novikova 1987).

*Procloeon maritimum* differs in the shape of maxillary palp, which is apically rounded and distinctly thicker in *P. maritimum* compared to *P. caspicum* sp. n. (figs 5, 20 in Bae and Park 1997). Moreover, length of the apical segment of maxillary palp reaches less than 1/3 the length of segment II (Bae and Park 1997), compared to approximately 1/2 in *P. caspicum* sp. n. Tarsal claws are slightly shorter in *P. maritimum*, reaching 0.38 × foretarsus length compared to 0.44 × in *P. caspicum* sp. n. and 0.45 × middle and hind tarsus length compared to 0.55 × in *P. caspicum* sp. n. (see Kluge 1983). The arrangement of the inner margin of paraproct also slightly differs, with a higher number of teeth of more irregular size occurring in *P. maritimum* (fig. 110 in Kluge 1983).

*Procloeon calabrum* can be reliably distinguished from *P. caspicum* sp. n. based on several characters. It differs in the shape of labrum, with medial notch on anterior margin much more pronounced in *P. caspicum* sp. n. compared to *P. calabrum*. Anterior margin laterally from the medial notch is symmetrically rounded in *P. calabrum* (fig. 9 in Belfiore and D’Antonio 1990), whereas it is strongly asymmetric in *P. caspicum* sp. n. (Fig. 4). Maxillary palps are only two-segmented in *P. calabrum*, contrary to a distinguishable third segment in *P. caspicum* sp. n. Another diagnostic character is represented by the length of tarsal claws (see Figs 12–14). In *P. calabrum*, tarsal claws in forelegs are equal to 3/4 of tarsi, in middle and hind legs hardly reaching 3/4 of tarsi (Belfiore and D’Antonio 1990). In *P. caspicum* sp. n., tarsal claws in all legs are distinctly shorter (see Figs 12–14). The egg chorion of *P. caspicum* sp. n. also lacks the equatorial band of large papillae, present in *P. calabrum*.

**List of species known from Iran.** A detailed review of literature revealed 42 references published in international journals accessible to the scientific public. Publications written in Persian (Farsi) were previously reviewed by Sharifinia (2015) and they did not include any species not reported in international sources reviewed (cf. Table 2 summarizing macroinvertebrate diversity in Sharifinia 2015). Despite relatively high number of recent (after 2000) publications on macroinvertebrates based on routine sampling of benthic communities, the knowledge on aquatic diversity seems to be very limited. Most studies include data on macroinvertebrates determined to family level (e.g., Nemati Varnosfaderany et al. (2010), Montajami et al. 2012, Abbaspour et al. 2013, Bashti and Ostovan 2014, Eyidozehi et al. 2014, Nasirian 2014, Aazami et al. 2015, Shayeghi et al. 2016) or generic level only (e.g., Egglishaw 1980, Mousavi Nadushan and Ramezani 2011, Mahboobi Soofiani et al. 2012, Imanpour Namin 2013, Ghasemi and Kamali 2014, Seyyedsharifi et al. 2014, Shokri et al. 2014, Shayeghi et al. 2015, Sharifinia et al. 2016a,b), reporting predominantly common Palaearctic families and genera. Unfortunately, determination to species level (if present) is erroneous in most cases in question. Altogether 27 records of species or genera (Table 2, comments below) with restricted distribution to the Nearctic and Neotropic Region are listed, suggesting that the authors used inappropriate determination keys. For example, the listed Nearctic/Neotropic genus *Lachlania* in fact most likely represents *Oligoneuriella* that is widely distributed in north Iran (cf. Table 2); the same concerns the Nearctic/Neotropic genus *Campsurus* which in fact most probably represents *Ephoron*. Likewise, the Nearctic/Neotropic genus *Callibaetis* could refer to cosmopolitan *Cloeon*, and the Nearctic/Neotropic genus *Tricorythodes* seems to refer to cosmopolitan *Caenis*, etc. A review of macroinvertebrates of Iranian running waters by Sharifinia (2015), despite promising “critical re-identification of the reported species”, includes such confusing data not only in mayflies, but also in Plecoptera. Therefore, we do not recommend using this list as reliable and valid source of information on the diversity of Iranian benthic insects. Relevant information on mayfly diversity was only found mainly in 20^th^ century publications in international entomological journals. However, these are highly fragmented and refer to material often limited to occasional collections with only several specimens examined. Moreover, these records are almost completely confined to the northern part of Iran, mostly Alborz Mts.

Broadening literature data with new material sampled in 2016, we conclude altogether 48 species records and 22 records at generic/subgeneric level of determination (Table 2). Records of Nearctic/Neotropic species and genera were excluded. We included all records of species and genera distributed in the Palaearctic Region, although we regard the occurrence of seven of them as doubtful. This concerns species/genera which have never been recorded at such low latitude (*Ameletus*, *Arthroplea*, and *Leptophlebia*) and so easternmost (*Siphlonurus*, *Ephemerella maculocaudata*) or westernmost (*Cinygmula*, *Baetis bicaudatus*) in the West Palaearctic Region. The genus *Arthroplea*, although exhibiting some southern area disjunctions in Europe, is predominantly boreal (Bauernfeind and Soldán 2012) and thus, its occurrence in the Middle East could be excluded. The genus *Leptophlebia* shows similar distribution as *Arthroplea* (although not so strictly boreal) in the West Palalearctic Region and is missing even in eastern Mediterranean and Caucasus. The occurrence of *Ephemerella maculocaudata* in Iran is very unlikely as its easternmost records are from the Balkans (Bulgaria and Macedonia). This record most probably refers to the recently described *Serratella elissa*, as its larvae similar to *E. maculocaudata* exhibit few basal dark brown segments of cerci (cf. Soldán 1982, Jacobus et al. 2009). Moreover, *S. elissa* is very common and abundant in the coastal area of the Caspian Sea and the type locality of *S. elissa* is about 150 km far from the locality of *E. maculocaudata*. The remaining four doubtful records are not fully improbable and need to be confirmed. The genus *Siphlonurus*, common in Europe, Far East and Japan, is very sparsely distributed in eastern Turkey and western Caucasus, but missing in the Middle East countries and Central Asia (Bauernfeind and Soldán 2012). The genus *Ameletus* is widely distributed in Europe, Siberia, Central Asia and Far East, however, its southern area border is insufficiently known (Bauernfeind and Soldán 2012). It occurs in Turkey but is missing in the Caucasus. The western limit of the areas of the genus *Cinygmula* and *Baetis bicaudatus* is in Central Asia and Mongolia, respectively (Bauernfeind and Soldán 2012). Moreover, the genus *Cinygmula* can be easily confused with the genus *Rhithrogena*.

Excluding *B. bicaudatus* and *E. maculocaudata* as discussed above, 46 reliable species were recorded, 18 species of them were recorded to Iran for the first time (in bold in Table 2). These species can be classified into the following groups from the biogeographical point of view.

(i) Holarctic and Transpalaearctic species form the minority of the mayfly fauna of Iran, encompassing six eurytopic species: *Baetis fuscatus*, *Baetis rhodani*, *Cloeon simile*, *C. cognatum*, *C. dipterum*, and *Serratella ignita*. Concerning the genus *Cloeon*, there are persisting taxonomic and determination problems, especially in the subgenus Cloeon s. str. and actual findings in Iran, thus, should be considered with caution. Likewise, *B. rhodani* is currently considered a polytypic species with the cryptic species throughout the geographical range (Williams et al. 2006).

(ii) West Palaearctic species with southern area limit in the Middle East included 13 species. Most of them are widely distributed throughout the whole area: *Baetis buceratus*, *B. lutheri*, *B. nexus*, *B. vardarensis*, *Paraleptophlebia submarginata*, *Habroleptoides confusa*, *Habrophlebia lauta*, *Ephemera danica*, *Palingenia longicauda*, and *Caenis macrura* (Bauernfeind and Soldán 2012). Iran is the natural south eastern area limit for many West Palaearctic species since the Caspian Sea, arid areas in central Iran, and large deserts in east Iran are the barriers separating Central Asia and the Indian subcontinent. Three species, *Palingenia fuliginosa*, *Heptagenia samochai* and *Epeorus zaitzevi*, show a peculiar central Palaearctic distribution, missing in central, northern and western Europe. *P. fuliginosa* shows an arc-like area spreading from eastern Slovakia and Ukraine to Caucasus and Caucasian part of north Iran (Soldán 1978b, Bauernfeind and Soldán 2012). *Heptagenia samochai* is distributed in Israel and from the Crimean Peninsula and Transcaucasia to Iran (Bauernfeind and Soldán 2012). *E. zaitzevi* is known from several Middle Eastern countries (Israel, Iraq, Syria, and Turkey) and from the Caucasus (Azerbaijan and Armenia) (Kluge 1997b, Bauernfeind and Soldán 2012).

(iii) West Palaearctic species with area disjunction to Central Asia, *Baetis gracilis* to Tajikistan, *B. muticus* to Kazakhstan (Bauernfeind and Soldán 2012), and *Baetis braaschi* distributed continuously from the Eastern Ukraine, Crimea and Caucasus Mts. through Iran and Turkmenistan to Central Asia (Sroka et al. 2012).

(iv) Caucasian species with the distribution reaching Alborz Mts. and Azerbaijan Provinces in north Iran: *Baetis ilex*, *B. vadimi*, *Oligoneuriella tskhomelidzei*, *Ecdyonurus ornatipennis*, *Electrogena pseudaffinis*, *E. squamata*, *Rhithrogena decolorata*, *Epeorus znojkoi*.

(v) Near and Middle East species include those described and known from Iran only: *Procloeon caspicum* sp. n., *Electrogena bothmeri*, *Rhithrogena iranica*, *R. paulinae*, *Epeorus caucasicus iranicus*, and *Serratella elissa*. Most of them are insufficiently known; *E. bothmeri* and *R. iranica* were described based on imagines (subimagines) only, the status of the latter species should be revised. The same concerns *Electrogena ressli* described from Turkey with paratypes from Gilan Province in Iran. On the contrary, only larvae were described in *E. caucasicus iranicus*, *S. elissa* and *P. caspicum* sp. n. Real distribution of all these species is unknown. Other species are, beside Iran, known from a single neighbouring country: *Baetis baroukianus* (Lebanon), *Baetis monnerati* (Jordan), *Choroterpes sumbarensis* and *Caenis kopetdagi* (Turkmenistan), *Teloganopsis subsolana* (Afghanistan), *Mortogenesia mesopotamica* (Iraq), *Palingenia orientalis* (Israel), and *Clypeocaenis bisetosa* (India). The only exception is *B. samochai* which inhabits the whole Near East (Turkey, Israel, Lebanon, Syria, and Iran).

Most studies and records on mayflies are available from northern Iran which belongs to Euxino-Hyrcanian Province of the Euro-Siberian subregion of the Palaearctic Region (Sagheb Talebi et al. 2014). They provide a good example of species of West Palaearctic (or European) origin with eastern area limits in Iran. Additionally, the Caucasian faunistic elements are reaching eastwards the northern mountains (Alborz Mts., Talysh Mts., Arasbaran Mts. and their foothills). Future detailed research will probably reveal a closer relation to the Caucasus bioregion and simultaneously, some endemic species could be expected there. This region is exceptional and attractive for scientists due to the Hyrcanian Forest, which is the hot spot of biodiversity of flora and fauna (Tohidifar et al. 2016). The Caspian Hyrcanian Forest in Iran and Azerbaijan is among the last extensive relicts of temperate primeval forests in the world hosting diverse insect specialists that are extinct in Europe and other parts of the world (see Müller et al. 2015, 2017). In contrast, knowledge on mayflies of the large area of central Iran, biogeographically belonging to the Irano-Turanian Province of the Central Asian subregion, is insufficient. This area includes arid and desert Central Plateau and large mountain range of Zagros Mts., which hardly ever were investigated. Local endemic species restricted to isolated or relict aquatic biotopes can hypothetically be discovered in this region. The southernmost part of Iran belongs to the Saharo-Sindian Province of the Euro-Siberian subregion, which covers also several other Middle East countries, such as neighbouring Iraq, part of Saudi Arabia and Syria. The occurrence of faunistic elements from the western part of this Province (Arabian Peninsula and North Africa) in the southern Iran can be hypothesized. Unfortunately, there are no data on mayflies from southern Iran to date.

This list of Ephemeroptera of Iran is undoubtedly preliminary and incomplete due to limited literature sources and lack of correct determination of material collected for water quality assessment. Thus, the total number of 46 species recorded is very low and does not represent the real diversity of mayflies in Iran. In comparison, Odonata, a very attractive and popular group of aquatic insects, have been better investigated at least from the faunistic point of view, with records of 100 species and subspecies throughout Iran (see current check list by Heidari and Dumont 2002 and many recent studies: Ebrahimi et al. 2009, Sadeghi and Mohammadalizadeh 2009, Ghahari et al. 2009, 2012, Eslami et al. 2014, 2015, Kiany and Sadeghi 2016). Likewise, faunistic records of Trichoptera include 130 species (see current check list by Mirmoayedi and Malicky 2002 and some important recent studies: Malicky 2004, Mey 2004, Chvojka 2006), pointing at the real diversity of the area. Comparatively less is known about Iranian stoneflies, which were studied in detail only in the northern part of the country (Aubert 1964, Murányi 2005), or aquatic beetles (e.g., Olmi 1981, Vafaei et al. 2007, 2008, 2009, Ghahari and Jedryczkowski 2011, Ghahari et al. 2015, Jäch et al. 2016). However, the distribution and diversity of all these aquatic groups were investigated predominantly based on their adults and/or terrestrial stages. Larvae of many species have not been described yet and, consequently, virtually nothing is known on their biology and ecological requirements.

To fill evident gaps in our knowledge resulting from this review, we aim to work on a more extensive study of Iranian Ephemeroptera covering the geographical gradients within Iran. This may unravel unknown species and diversity in different biogeographical provinces of Iran. This however would require to set up a network of localities and to study at least some of them in different seasonal aspects. Our first field trips to Iran in 2016 and 2017, however, showed us that aquatic ecosystems have been under strong, long-term anthropogenic pressure and some areas unfortunately presumably no longer maintain their original aquatic biodiversity. We observed many rivers with severe pollution that most probably wiped out local populations of the aquatic fauna. Overexploitation of water sources and growing pollution from fertilisers, pesticides and municipal and industrial wastewaters are serious threats to aquatic biodiversity. Iran has 7.2 million ha of agriculture land dependent on irrigation, the largest area in the Middle East, thus, agricultural use accounts for more than 90% of total water withdrawal. About 1.7 million ha of irrigated land is affected by salinization (World Commission on Dams 2000, Afkhami 2003). About 96 % of the urban population of Iran is connected to public water supplies; however, only 16 % are connected to adequate sewage treatment facilities (see Charkhabi et al. 2005, Afkhami et al. 2007). There are also significant problems caused by insufficient treatment of industrial wastewaters leading to serious impacts of heavy metals and other toxic compounds (e.g., Gheshlagh et al. 2013, Khodadadi et al. 2013, Mollazadeh et al. 2013, Majnoni et al. 2015) which affect, beside aquatic ecosystems, also human health (e.g., Karrari et al. 2012). Moreover, the absence of real regulations of water abstraction from rivers and lakes and obligatory minimal flows from impoundments seriously impacts hydrology of streams and their ecosystem functioning. It underlines the importance to study both regional and local aquatic diversity until it totally disappears. The discovery of possible refugia for aquatic biota, which should be proposed as priority for immediate conservation, is an urgent goal to preserve the aquatic biodiversity of Iran. However, only thorough basic taxonomic and faunistic research can contribute to water and conservation management set by the local authorities.

## Supplementary Material

XML Treatment for Procloeon (Pseudocentroptilum) caspicum
